# Association of Centre Quality Certification with Characteristics of Patients, Management, and Outcomes Following Carotid Endarterectomy or Carotid Artery Stenting

**DOI:** 10.3390/jcm13154407

**Published:** 2024-07-28

**Authors:** Stefan Saicic, Christoph Knappich, Michael Kallmayer, Felix Kirchhoff, Bianca Bohmann, Vanessa Lohe, Shamsun Naher, Julian Böhm, Sofie Lückerath, Hans-Henning Eckstein, Andreas Kuehnl

**Affiliations:** 1 Department for Vascular and Endovascular Surgery, University Hospital rechts der Isar, Technical University of Munich, 81675 Munich, Germany; stefan.saicic@mri.tum.de (S.S.);; 2 Landesarbeitsgemeinschaft zur Datengestützten, Einrichtungsübergreifenden Qualitätssicherung in Bayern (LAG Bayern), 80331 Munich, Germany

**Keywords:** carotid stenosis, carotid endarterectomy, carotid stenting, quality assurance, quality certification

## Abstract

**Background:** The aim of this study was to analyze the association between center quality certifications and patients’ characteristics, clinical management, and outcomes after carotid revascularization. **Methods:** This study is a pre-planned sub-study of the ISAR-IQ project, which analyzes data from the Bavarian subset of the nationwide German statutory quality assurance carotid database. Hospitals were classified as to whether a certified vascular center (cVC) or a certified stroke unit (cSU) was present on-site or not. The primary outcome event was any stroke or death until discharge from the hospital. **Results:** In total, 31,793 cases were included between 2012 and 2018. The primary outcome rate in asymptomatic patients treated by CEA ranged from 0.7% to 1.5%, with the highest rate in hospitals with cVC but without cSU. The multivariable regression analysis revealed a significantly lower primary outcome rate in centers with cSU in asymptomatic patients (aOR 0.69; 95% CI 0.56–0.86; *p* < 0.001). In symptomatic patients needing emergency treatment, the on-site availability of a cSU was associated with a significantly lower primary outcome rate (aOR 0.56; 95% CI 0.40–0.80; *p* < 0.001), whereas the presence of a cVC was associated with higher risk (aOR 3.07; 95% CI 1.65–5.72). **Conclusions:** This study provides evidence of statistically significant better results in some sub-cohorts in certified centers. In centers with cSU, the risk of any stroke or death was significantly lower in asymptomatic patients receiving CEA or symptomatic patients treated by emergency CEA.

## 1. Introduction

According to international carotid guidelines, carotid endarterectomy (CEA) is considered the treatment of choice for patients with 60–99% asymptomatic or 50–99% symptomatic carotid artery stenosis [1,2,3,4]. Carotid artery stenting (CAS) may be considered a less invasive treatment option, especially if the patient is at high risk for surgery [5,6]. In addition, factors such as the degree of stenosis, preceding radiation therapy, or symptomatic status are considered when choosing a treatment procedure between CEA and CAS [4,7]. Previous large studies comparing the outcomes of CAS and CEA demonstrated varying results, with the majority showing a higher risk of periprocedural stroke in CAS compared to CEA, while others found no major difference with regard to the long-term outcomes [8,9,10,11,12,13,14,15,16,17,18,19]. Data suggesting a higher risk of periprocedural outcomes in symptomatic patients, even with lower grades of stenosis, focus attention on the importance of plaque morphology and individual patient characteristics for preoperative risk assessment [10]. Regarding hospital-level characteristics, an inverse association between annual hospital volume and the risk of in-hospital stroke after carotid revascularization has already been demonstrated [20,21,22]. Additionally, there is also evidence showing high-volume vascular surgeons achieving significantly lower perioperative stroke or death rates after CEA [23]. Explanations for the connection between high volume and better outcomes often refer to better experience or ‘routine’ on the part of medical staff as well as optimized structures and processes.

The aim of quality certifications, according to the German Society for Vascular Surgery (DGG), is to improve patient care quality measures by optimizing structural quality and process quality, e.g., minimum requirements for staff qualification, multidisciplinary team settings, or quality assurance measures within the framework of an economically oriented healthcare system. Criteria for a cVC include, among others, on-site availability of units for vascular surgery, radiology, and angiology, as well as a minimum annual caseload of more than 30 CEA [24]. Patient treatment should be optimized by close cooperation with departments of, e.g., neurology and cardiology and regular conduction of mortality and morbidity conferences. The German Stroke Society (DSG) defines similar strict criteria for cSU, including a 24 h availability of personnel performing computed tomography and recanalizing interventions [25]. So far, the impact of center quality certifications, e.g., availability of stroke units (cSU) or certified vascular centers (cVC), on the management and outcome after CEA or CAS has not been investigated.

Therefore, the aim of this study was to analyze the association between center quality certification and characteristics of patients, clinical management, and outcomes after carotid revascularization by CEA or CAS. 

## 2. Methods

The present analysis is a pre-planned sub-study of the ISAR-IQ project (Integration and Spatial Analysis of Regional, Site-Specific, and Patient-Level Factors for Improving Quality of Treatment for Carotid Artery Stenosis). The methods of the ISAR-IQ project have already been published elsewhere [26,27] and are summarized here.

### 2.1. Data Source

The study is based on the Bavarian subset of the German nationwide statutory quality assurance database operated by the Bavarian Institute for Quality Assurance (former: Bayerische Arbeitsgemeinschaft für Qualitätssicherung in der stationären Versorgung, BAQ) [28]. Data from quality assurance measures according to § 136 SGB V of the Federal Joint Committee were used. Because of legal obligations [29], the data collection covers all CEA operations and CAS procedures. All hospitals in Germany have to report the details of carotid procedures in this database by law. The data and results, therefore, did not have to be collected first for this study but were available in the form of a completed database that was used for secondary data analysis. The legal basis for the scientific use of quality assurance data is § 137a paragraph 10 SGB V. The study was approved by the ethics committee of the Medical Faculty, Technical University of Munich (Reference Number 107/20S). The analysis was conducted according to Good Practice of Secondary Data Analysis guidelines [30]. As this is an observational study using routinely collected health data, RECORD reporting guidelines were applied [31]. All data were saved on BAQ servers according to the respective data protection regulations. Data access was only permitted using controlled data processing (CDP). Essential methods regarding CDP have been established in other studies and reported [26,27,32,33,34,35]. The study protocol was submitted to the BAQ and the G-BA during the application procedure but was not published separately. Further details of the legal basis have already been published [32].

### 2.2. Case Selection

Symptomatic and asymptomatic patients who underwent elective CEA and CAS between January 2012 and December 2018 were included in the analysis (for patients’ flowcharts, see Supplemental Figure S1). The indication groups were defined as follows: group A, asymptomatic cases; group B, symptomatic cases electively treated for amaurosis fugax, transitory ischemic attack, stroke, or other symptoms; group C1, patients suffering crescendo-TIA or stroke-in-evolution; group C2, CEA or CAS in the context of simultaneous procedures. Simultaneous procedures considered were aorto-coronary bypasses, peripheral arterial reconstructions, aortic reconstructions, and intracranial PTA/stenting.; group C3, CEA, or CAS for other indications (e.g., carotid aneurysm, symptomatic coiling, redo carotid procedures, tandem stenosis). Patients not fitting these criteria were excluded from the analysis.

### 2.3. Study Variables

The main variable of this study was center quality certification, coded in two variables (cVC certified by the German Vascular Society): yes or no, and availability of a certified stroke unit (cSU certified by the German Stroke Society): yes or no. This resulted in four hospital groups for cVC/cSU: yes/yes, yes/no, no/yes, and no/no. 

### 2.4. Study Outcomes

The primary outcome event (POE) was defined as any stroke or death until discharge from the hospital. Secondary outcomes were defined as major stroke or death, any stroke, all-cause death, myocardial infarction (MI), and major adverse cardiovascular event (MACE), all until hospital discharge. All outcomes were subdivided into the aforementioned indication groups (A, B, C1–3). In the report form for the quality assurance database, the hospitals have to specify the preoperative symptom status and can provide this information by selecting the following symptoms: amaurosis fugax, transitory ischemic attack, stroke (including severity by use of the modified Rankin Scale) or “other neurological features”. Regarding the outcome, the occurrence of a new perioperative neurological event (including severity by use of the modified Rankin Scale) must be reported in the database.

### 2.5. Statistical Analyses

Categorical variables were shown as absolute numbers and percentages. If not stated otherwise, continuous variables were presented as median with first (Q1) and third (Q3) quartiles. To account for confounding, the variables age, sex, American Society of Anesthesiologists (ASA) stage, type of index event in symptomatic patients (i.e., amaurosis fugax, TIA, minor stroke, major stroke), ipsilateral and contralateral degree of stenosis, pre- and post-procedural assessment by a neurologist, and hospital volume were entered as fixed-effect factors. Model specification and variable selection were conducted a priori according to a prespecified analysis plan based on literature research and expert knowledge. 

R version 3.2.1 (R Foundation for Statistical Computing, Vienna, Austria [36]) was used for data processing and statistical analysis, with extension packages *tidyverse* and *epitools* to calculate cross-classified tables, chi-square tests, and multivariable regression analyses. Variable codes were extracted from the codebooks provided by the BAQ and harmonized over the time period from 2012 to 2018. For all tests, a two-tailed level of significance of α = 5% was used.

## 3. Results

### 3.1. Baseline Characteristics

In total, 31,793 cases were included. The majority of patients (23,199, 73%, Table 1) were treated in a non-certified vascular center, with 35% being treated in a center with cSU. In total, 27% of patients were treated in a cVC with 16% of these patients being treated in a center with an on-site cSU. The majority of patients were male (68%), and the median age was 72 years (Q1/3: 65–78). Most patients were classified ASA III (62%). In hospitals with both certified centers, most patients (91%) presented with severe carotid stenosis (70–99%, NASCET). About two-thirds of the cases were asymptomatic, with the lowest share in centers with cSU but without cVC (49%). Across all centers, high availability of vascular surgeons was observed, with the lowest being in hospitals without cVC (94–96%, Table 2). Higher variability could be found in the availability of neurologists, with the highest being in cVC with cSU (97%) and the lowest in centers without either (43%). Neuroradiologists showed the highest availability in cVC with a small difference in centers with cSU (yes: 76%, no: 77%). In centers without any certification, neuroradiologists were available in 25% of centers.

Analysis of the center’s annual caseload showed a total of 94 CEA performed in centers with cSU and cVC, with 14 CEA being performed in centers without cSU and cVC. Regarding urban-rural differences, hospitals with double certification were predominately situated in large independent cities (60%, Table 2).

### 3.2. Diagnostic Procedures, Management, and Treatment

In all indication groups, a higher proportion of patients was treated with CEA compared to CAS, Table 3. The proportion of patients with pre- and post-procedural neurological assessments was higher in hospitals with cSU (68% and 54%) compared to hospitals without cSU (40% and 31%). In all hospitals, the majority of patients received perioperative antiplatelet medication with Aspirin, with the highest proportion in cVC with cSU (89%). In the case of CEA, local anesthesia was more frequently used in cSU (26–30%). The rate of combined/converted anesthesia was lowest in hospitals with a cVC (0.4–1.3%).

### 3.3. Study Outcomes

The primary outcome rate in asymptomatic patients treated by CEA ranged from 0.7% to 1.5%, with the highest rate in hospitals with cVC but without cSU (Table 4). Regarding symptomatic patients (non-emergency) treated by CEA, the primary outcome risk ranged from 1.8% to 2.7%. See Table 4 for details regarding primary outcome rates and Supplemental Tables S1 and S2 for secondary outcomes. 

**Figure 1:**
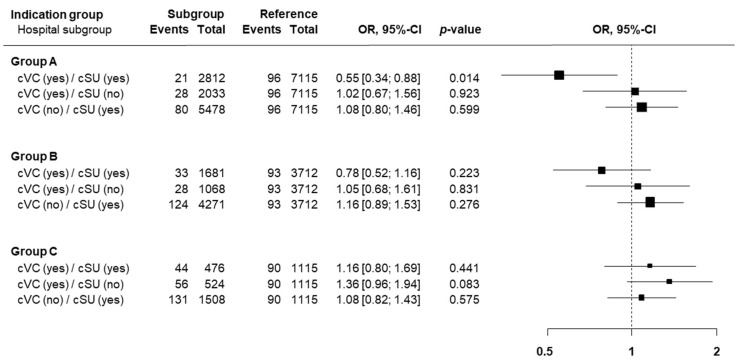
Univariate analysis of the association with the primary endpoint. Indication group A: asymptomatic patients. Indication group B: symptomatic patients receiving elective CEA or CAS. Indication group C: Patients treated by CEA or CAS for other indications. OR: odds ratio, cVC = certified vascular center, cSU = certified stroke unit. Reference = center without cVC or cSU.

In the univariate analysis (Figure 1), the in-hospital risk of stroke or death for asymptomatic patients was significantly lower in double-certified centers (OR 0.55; 95%-CI 0.34–0.88, *p* = 0.014) compared to centers without cSU or cVC.

**Figure 2:**
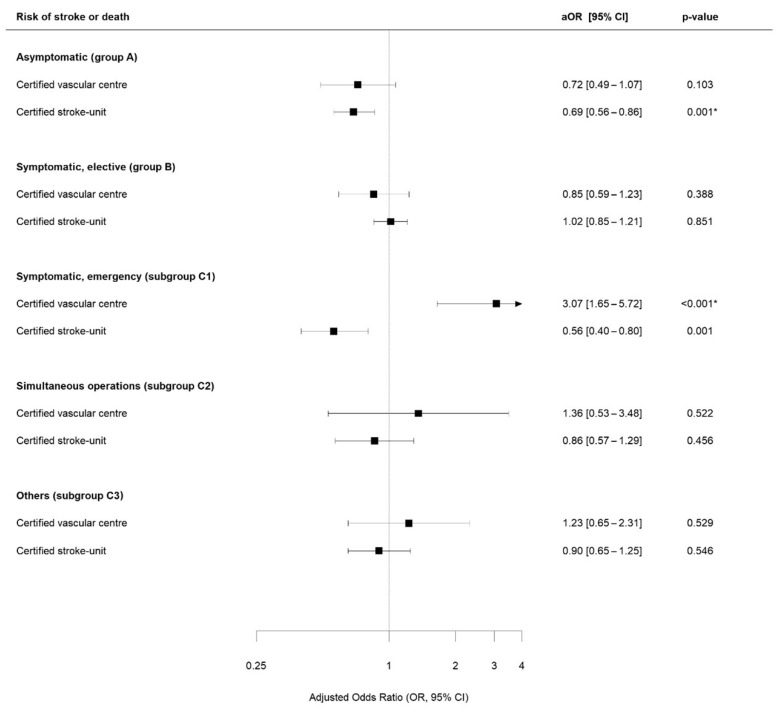
Multivariable regression analysis for patients treated with CEA. aOR = odds ratio for the primary endpoint adjusted for age, sex, ASA stage, degree of stenosis ipsilateral and contralateral stenosis, pre- and post-procedural neurological examination, annual hospital volume (log-transformed), time-interval between index event and treatment (only group B), and neurological symptoms (only symptomatic patients). Reference = no on-site certified vascular center or stroke unit. * = statistical significance (*p* < 0.05).

**Figure 3:**
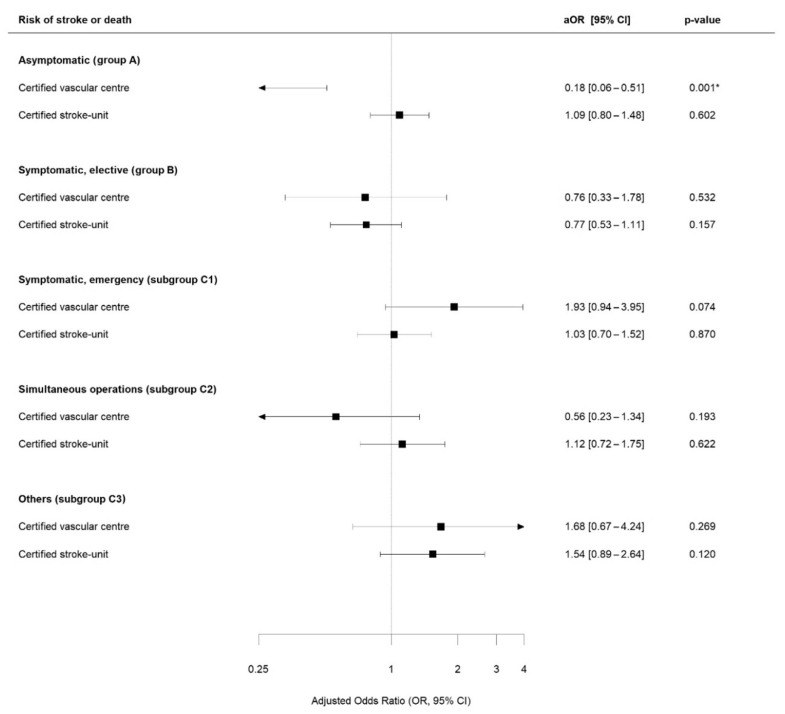
Multivariable regression analysis for patients treated with CAS. aOR = odds ratio for the primary endpoint adjusted for age, sex, ASA stage, degree of stenosis ipsilateral and contralateral stenosis, pre- and post-procedural neurological examination, annual hospital volume (log-transformed), time-interval between index event and treatment (only group B), and neurological symptoms (only symptomatic patients). Reference = no on-site certified vascular center or stroke unit. * = statistical significance (*p* < 0.05).

The multivariable regression analysis revealed a significantly lower primary outcome rate in centers with cSU in asymptomatic patients treated with CEA (aOR 0.69; 95%-CI 0.56–0.86; *p* < 0.001). In symptomatic patients treated with CEA needing emergency treatment (indication group C1), the presence of a cSU was associated with a significantly lower primary outcome rate (aOR 0.56; 95%-CI 0.40–0.80; *p* < 0.001), whereas the presence of a cVC was associated with a higher risk (aOR 3.07; 95%-CI 1.65–5.72). See Figure 2 for further details on CEA outcomes and Figure 3 regarding primary outcomes following CAS.

## 4. Discussion

The present study is based on a statutory state-wide Bavarian full survey and, to our knowledge, is the first study to analyze the influence of certified care structures in the treatment of carotid stenosis. In general, this study provides evidence of statistically significant better results in some sub-cohorts in certified centers, but there is no evidence of clinically relevant and comprehensive care deficits in clinics without either certification. In particular, the availability of an on-site cSU was associated with a significantly lower risk of any stroke or death following CEA in asymptomatic patients and symptomatic emergency patients with crescendo-TIA or stroke in evolution. Regarding CAS, on-site availability of a cVC was associated with a significantly lower risk of stroke or death in asymptomatic patients. 

A main result of the present analysis was that CEA in asymptomatic patients was independently associated with lower in-hospital rates of stroke or death when performed in centers with cSU. However, CAS in asymptomatic patients was associated with a lower risk when performed in centers with cVC. This could be due to various factors, such as a higher annual volume and thus more experienced interventionalists, optimized processes, better patient management, or a higher ability to rescue. This might also be due to more precise indication for treatment and better method selection between CEA and CAS after multidisciplinary team review as recommended by international guidelines [2]. Unfortunately, all these factors are not documented in the nationwide legally defined quality assurance database and could, therefore, not be included in this analysis. Both CEA and CAS are procedures requiring sophisticated surgical or interventional skillsets and should ideally be performed by experienced practitioners in hospitals that are accustomed to performing the respective procedure on a regular basis. The performance of CEA and CAS requires the availability of hospital specialists with the appropriate qualifications. As presented in Table 2, almost every CEA was performed in a hospital with the availability of a vascular surgeon, but only 25% to 77% of CAS were performed in hospitals with a neuroradiologist. Unfortunately, we do not have detailed data on the individual interventions and the respective treating specialists, but only data on the availability of specialists in the individual hospitals. The training of specialists for CEA and CAS is not comparable, as CEA is performed by surgeons (vascular surgery, general surgery, neurosurgery), while CAS is mainly performed by interventionalists from radiology/neuroradiology or cardiology/angiology. Thus, our analysis cannot address how the surgeons/interventionalists were trained, as the data did not have to be recorded by the reporting hospitals. However, it can be assumed that CEA and CAS are performed in the majority of cases by differently trained physicians.

Hospitals with cVC and cSU probably have structural and personnel means for in-house training, vascular specialists, and a higher annual caseload compared to less specialized centers. Although multivariable regression analysis adjusted for hospital-level annual caseload, this observation might be traced back to individual surgeon experience, which is unfortunately not documented in the underlying database. For CEA, several studies have shown the performing surgeon’s specialty and case volume to have a significant effect on perioperative stroke and death risk [21,22,23,38,39]. Similar results, although not for hospital-linked CAS volumes, were found to be true for operator-linked CAS volumes [20]. The higher annual caseload was found in hospitals with certified structures, with the highest annual center volume in hospitals with both cSU and cVC (median 94), and the lowest annual center caseload in hospitals without any center certification (median 14). Particularly with regard to the endpoint death, hospitals with certified centers and higher annual volume probably also had more specialized departments and, thus, presumably better ability to rescue. However, on the basis of quality registry data, this analysis cannot prove what causes what and whether inverse causation is also involved.

An association between favorable outcomes and the availability of a cSU was found for CEA but not for CAS. The effect was strongest in group C1 (symptomatic, emergency cases), which may indicate the importance of perioperative and postoperative neurological patient monitoring and treatment. A possible explanation for this effect not being observed for CAS could be due to the narrow indication window for revascularization, with CAS showing higher risk during the first hours after symptom onset [40,41,42]. However, studies have shown that CAS performed by trained vascular surgeons significantly improves the outcome considering postoperative stroke and hospital readmission [43]. Overall, median caseloads for all hospitals considering CAS were lower than CEA. This might be explained by pointed guideline recommendations for CAS or local/individual preferences. Furthermore, the performance of complex stenting procedures requires specially trained personnel with appropriate equipment, which is more likely available in larger (certified) centers. Additionally, financial incentives regarding the DRG reimbursement system for stroke units, which are missing for vascular centers, could explain center distribution in favor of cSU and, thus, better personnel and material resources. 

In summary, this study provides evidence of statistically significant better results in some sub-cohorts in certified centers, but there is no evidence of clinically relevant and comprehensive care deficits in clinics without either certification. 

## 5. Limitations

This is a secondary data analysis, and thus, all shortcomings of observational studies using routine data must be considered as previously reported [21,32]. First, no information on the cause of death is provided in the registry. Second, the study design was retrospective and observational [44]. Because patients were not randomized for the different hospital groups, selection bias as well as confounding by indication is possible [45]. This implies that all results need to be interpreted as associations rather than causal relationships. Third, follow-up data covered only the in-hospital period. Because most of the perioperative events presumably occur within the first days after CEA or CAS, a detection bias is considered to be low [17]. Fourth, all data in the database are self-reported by the attending physicians, and reporting bias cannot be ruled out. However, data reports were reviewed by the regional offices for quality assurance (Landesgeschäftsstellen für Qualitätssicherung), and the occurrence of suspect data induced a process of so-called structured dialogue to clarify abnormalities systematically. Although under-reporting cannot be ruled out, any potential information bias is considered homogeneous among the variables analyzed in this study. Fifth, residual confounding cannot be excluded because some possible confounders were not collected (e.g., information on specific comorbidities, vascular anatomy, cardiovascular risk profile, routine medication, presence of restenosis, intraoperative heparin application or the reasons for the application or changes in a certain procedural technique). Sixth, only the degree of stenosis is given in the quality assurance database, while no detailed data on other sonographic features such as peak systolic velocity, plaque morphology, or calcification are included in the database [46,47]. 

Seventh, the German quality assurance database does not provide detailed information on comorbidities. The only variable related to comorbidity status that was available in the database and could, therefore, be analyzed was the preoperative stage in the American Society of Anesthesiologists (ASA) classification. ASA status was included in the multivariable regression to enable at least a basic risk adjustment. However, the inclusion of detailed information on patients’ comorbidities could have led to a more robust analysis.

## Figures and Tables

**Table 1:** Characteristics of patients treated with CEA or CAS by hospital-level quality structures.

Certified Stroke-Unit	Certified Vascular Centre							
	Yes				No			
	Yes		No		Yes		No	
**N (%)**	**4969**	**(16)**	**3625**	**(11)**	**11,257**	**(35)**	**11,942**	**(38)**
**Age (years, median, Q1–Q3)**	72	(65–77)	72	(65–78)	72	(65–78)	73	(66–78)
**Sex, Male**	3434	(69)	2443	(67)	7652	(68)	8120	(68)
**Right carotid artery treated**	2543	(51)	1795	(50)	5622	(50)	6115	(51)
**ASA stage**								
Stage I + II	1739	(35)	1224	(34)	3952	(35)	3734	(31)
Stage III	2937	(59)	2211	(61)	6675	(59)	7777	(65)
Stage IV + V	275	(5.5)	135	(3.7)	496	(4.4)	344	(2.9)
**Ipsilateral degree of stenosis**								
Mild (<50%, NASCET)	113	(2.3)	51	(1.4)	232	(2.1)	217	(1.8)
Moderate (50–69%, NASCET)	280	(5.6)	167	(4.6)	656	(5.8)	503	(4.2)
Severe (70–99%, NASCET)	4499	(91)	3333	(92)	10,041	(89)	11,094	(93)
Occlusion (100%)	77	(1.5)	74	(2.0)	328	(2.9)	128	(1.1)
**Contralateral degree of stenosis**								
Mild (<50%, NASCET)	3385	(68)	2350	(65)	7977	(71)	8012	(67)
Moderate (50–69%, NASCET)	683	(14)	645	(18)	1452	(13)	1860	(16)
Severe (70–99%, NASCET)	620	(12)	415	(11)	1194	(11)	1429	(12)
Occlusion (100%)	281	(5.7)	215	(5.9)	634	(5.6)	641	(5.4)
**Indication Group**								
- Group A (asymptomatic)	2812	(57)	2033	(56)	5478	(49)	7115	(60)
- Group B (symptomatic, elective)	1681	(34)	1068	(29)	4271	(38)	3712	(31)
Amaurosis fugax	258	(5.2)	214	(5.9)	576	(5.1)	579	(4.8)
Transitory ischemic attack (TIA)	589	(12)	429	(12)	1259	(11)	1404	(12)
Stroke (Rankin 0–5)	765	(15)	345	(9.5)	2245	(20)	1524	(13)
Other symptoms	69	(1.4)	80	(2.2)	191	(1.7)	205	(1.7)
- Group C (others)	476	(9.6)	524	(14)	1508	(13)	1115	(9.3)
Crescendo-TIA/Stroke-in-evolution	135	(2.7)	348	(9.6)	664	(5.9)	495	(4.1)
Simultaneous procedures ^#^	139	(2.8)	66	(1.8)	382	(3.4)	197	(1.6)
Others °	202	(4.1)	110	(3.0)	462	(4.1)	423	(3.5)

Q1 = first quartile, Q3 = third quartile, ASA = American Society of Anaesthesiologists physical status classification system (n = 294 data is missing in total). ^#^ = simultaneous performed coronary bypass operation, peripheral arterial reconstruction, aortic procedure, intracranial stenting, and other simultaneous performed procedures. ° = carotid aneurysm, symptomatic coiling, exulcerated plaque morphology, ipsilateral carotid occlusion, redo carotid procedures, tandem stenosis. NASCET = North American Symptomatic Carotid Endarterectomy Trial degree of stenosis criteria [37].

**Table 2:** Characteristics of patients regarding hospital characteristics.

Certified Stroke-Unit	Certified Vascular Centre							
	Yes				No			
	Yes		No		Yes		No	
**Specialists available at center**								
Vascular surgeon	4857	(98)	3615	(100)	10,557	(94)	11,427	(96)
Neurologist	4826	(97)	2882	(80)	10823	(96)	5109	(43)
Heart surgery	1843	(37)	3132	(86)	3811	(34)	4793	(40)
Internal medicine/Angiology	2575	(52)	2725	(75)	4656	(41)	5971	(50)
Internal medicine/Cardiology	4969	(100)	3489	(96)	10,866	(97)	10,524	(88)
Neurosurgery	4574	(92)	3058	(84)	8361	(74)	4799	(40)
Neuroradiology	3753	(76)	2794	(77)	5494	(49)	2932	(25)
**Certified Quality management system**								
DIN ISO EN 9001	1136	(23)	518	(14)	1441	(13)	2229	(19)
KTQ	159	(3.2)	144	(4.0)	1277	(11)	552	(4.6)
proCum Cert	0	(0.0)	0	(0.0)	0	(0.0)	526	(4.4)
none of these	3674	(74)	2963	(82)	8539	(76)	8635	(72)
**Centre annual caseload (median; Q1–Q3)**								
- All CEA	94	(76–120)	54	(37–98)	34	(10–65)	14	(3–35)
CEA in Group A	59	(34–74)	25	(15–58)	16	(2–32)	6.0	(1–21)
CEA in Group B	30	(26–45)	27	(14–38)	14	(3–26)	4.0	(1–12)
CEA in Group C	1	(0–4)	1	(0–3)	0	(0–1)	0	(0–1)
- All CAS	11	(3–29)	3	(1–20)	4	(0–19)	0	(0–4)
CAS in Group A	5.5	(2–11)	2.0	(0–4)	1.0	(0–5)	0.0	(0–2)
CAS in Group B	3.0	(0–6)	0.0	(0–2)	1.0	(0–6)	0.0	(0–1)
CAS in Group C	0	(0–2)	0	(0–1)	0	(0–1)	0	(0–0)
**Regional settlement structure**								
Independent city	2986	(60)	2836	(78)	5290	(47)	3849	(32)
Urban district	159	(3.2)	144	(4.0)	867	(7.7)	1251	(10)
Rural district	1170	(24)	463	(13)	1779	(16)	2557	(21)
Sparsely populated region	654	(13)	182	(5.0)	3321	(30)	4285	(36)

CEA = carotid endarterectomy, CAS = carotid artery stenting. Q1 = first quartile, Q3 = third quartile.

**Table 3:** Diagnostic procedures, management, and treatment of patients.

Certified Stroke-Unit	Certified Vascular Centre							
	Yes				No			
	Yes		No		Yes		No	
Time interval *								
0–2 days	243	(14)	186	(17)	643	(15)	376	(10)
3–7 days	627	(37)	437	(41)	1662	(39)	1398	(38)
8–14 days	282	(17)	149	(14)	652	(15)	722	(19)
15–180 days	460	(27)	228	(21)	889	(21)	1104	(30)
**Neurological assessment**								
Pre-procedural	3821	(77)	1941	(54)	8012	(71)	6903	(58)
Post-procedural	4151	(84)	1162	(32)	6918	(61)	5474	(46)
Pre- and post-procedural	3399	(68)	1107	(31)	6119	(54)	4732	(40)
**Perioperative antiplatelet medication**								
Aspirin monotherapy	3601	(72)	3225	(89)	8211	(73)	9431	(79)
Clopidogrel mono	128	(2.6)	44	(1.2)	244	(2.2)	329	(2.8)
Other monotherapy	9	(0.2)	13	(0.4)	76	(0.7)	31	(0.3)
Dual antiplatelet medication	715	(14)	250	(6.9)	1680	(15)	1524	(13)
None	516	(10)	93	(2.6)	1046	(9.3)	627	(5.3)
**Treatment by indication group**								
- Group A (asymptomatic)								
CEA	2427	(86)	1837	(90)	4592	(84)	6086	(86)
CAS	385	(14)	196	(10)	886	(16)	1029	(14)
- Group B (symptomatic, elective)								
CEA	1505	(90)	983	(92)	3367	(79)	3333	(90)
CAS	176	(10)	85	(8.0)	904	(21)	379	(10)
- Group C (others)								
CEA	296	(62)	413	(79)	723	(48)	932	(84)
CAS	180	(38)	111	(21)	785	(52)	183	(16)
**Type of anesthesia °**								
Local anesthesia	796	(26)	292	(15)	1710	(30)	1950	(23)
General anesthesia	2279	(73)	1608	(84)	3903	(68)	6218	(73)
Combined anesthesia	41	(1.3)	8	(0.4)	119	(2.1)	357	(4.2)
**Intraprocedural monitoring ^§^**								
Electroencephalography	46	(2.3)	135	(28)	150	(3.5)	207	(4.0)
Transcranial Cerebral Oximetry	631	(31)	53	(11)	538	(13)	824	(16)
Somato-sensory evoked potentials	831	(41)	118	(24)	2026	(48)	2122	(41)
Other methods	497	(25)	184	(38)	1518	(36)	2063	(40)

* = Time interval between the index event and time of treatment (only for Group B). ° = only CEA from 2012–2016. ^§^ = if documented.

**Table 4:** Primary outcomes in patients treated with carotid endarterectomy (CEA) or carotid stenting (CAS).

Certified Stroke-Unit	Certified Vascular Centre							
	Yes				No			
	Yes		No		Yes		No	
**Carotid endarterectomy (CEA)**								
**Any stroke or death**								
- Group A: asymptomatic	18/2427	(0.7)	27/1837	(1.5)	47/4592	(1.0)	74/6086	(1.2)
- Group B: symptomatic, elective	27/1505	(1.8)	24/983	(2.4)	92/3367	(2.7)	76/3333	(2.3)
- Group C1: symptomatic, emergency	5/64	(7.8)	27/299	(9.0)	16/299	(5.4)	40/430	(9.3)
- Group C2: Simultaneous procedures	6/72	(8.3)	5/35	(14)	14/159	(8.8)	11/124	(8.9)
- Group C3: Others	12/160	(7.5)	8/79	(10)	16/265	(6.0)	24/378	(6.3)
**Carotid artery stenting (CAS)**								
**Any stroke or death**								
- Group A: asymptomatic	3/385	(0.8)	1/196	(0.5)	33/886	(3.7)	22/1029	(2.1)
- Group B: symptomatic, elective	6/176	(3.4)	4/85	(4.7)	32/904	(3.5)	17/379	(4.5)
- Group C1: symptomatic, emergency	9/71	(13)	8/49	(16)	37/365	(10)	6/65	(9.2)
- Group C2: Simultaneous procedures	4/67	(6.0)	6/31	(19)	27/223	(12)	6/73	(8.2)
- Group C3: Others	8/42	(19)	2/31	(6.5)	21/197	(11)	3/45	(6.7)

Q1/3 = first/third quartile.

## Data Availability

The datasets analyzed during the current study are available on request from the IQTIG, https://iqtig.org/qs-verfahren-uebersicht/sekundaere-datennutzung/.

## Supplementary Materials

The following supporting information can be downloaded at https://www.mdpi.com/article/10.3390/jcm13154407/s1, Table S1: Secondary outcomes in patients treated with carotid endarterectomy (CEA). Table S2: Secondary outcomes in patients treated with carotid artery stenting (CAS). Table S3: Regional characteristics of treating hospitals. Figure S1: Patient flowchart.
