# Supplementary material for: Association of Centre Quality Certification with Characteristics of Patients, Management, and Outcomes Following Carotid Endarterectomy or Carotid Artery Stenting
Source: J Clin Med. 2024 Jul 28;13(15):4407. doi: 10.3390/jcm13154407 (PMC11313300; doi:10.3390/jcm13154407)
Supplement: Supplementary file 1 [file jcm-13-04407-s001.zip › jcm-3052805-supplementary.pdf]

Supplemental material:

Supplemental Table S1: Secondary outcomes in patients treated with carotid endarterectomy (CEA)

	**Certified vascular centre**			
	Yes		No	
**Certified stroke-unit**	Yes	No	Yes	No
**Group A: asymptomatic**				
Post-OP Hospital stay in days (M; Q1–Q3)	4 (3-5)	4 (3-4)	4 (4-5)	4 (4-5)
Major stroke or death	8 (0.3)	22 (1.2)	29 (0.6)	48 (0.8)
Any stroke	16 (0.7)	24 (1.3)	39 (0.8)	61 (1.0)
All-cause death	4 (0.2)	7 (0.4)	8 (0.2)	16 (0.3)
MI	3 (0.2)	3 (0.3)	7 (0.3)	12 (0.3)
MACE	14 (0.9)	14 (1.4)	28 (1.3)	51 (1.3)
**Group B: symptomatic, elective**				
Post-OP Hospital stay in days (M; Q1–Q3)	5 (3-6)	4 (3-6)	5 (4-6)	5 (4-6)
Major stroke or death	23 (1.5)	16 (1.6)	71 (2.1)	66 (2.0)
Any stroke	18 (1.2)	17 (1.7)	68 (2.0)	62 (1.9)
All-cause death	10 (0.7)	9 (0.9)	32 (1.0)	21 (0.6)
MI	4 (0.4)	3 (0.5)	6 (0.4)	4 (0.2)
MACE	13 (1.4)	16 (2.9)	44 (2.9)	39 (2.0)
**Group C1: symptomatic, emergency**				
Post-OP Hospital stay in days (M; Q1–Q3)	8 (4-11)	4 (4-7)	6 (4-9)	5 (4-8)
Major stroke or death	4 (6.3)	26 (8.7)	13 (4.3)	33 (7.7)
Any stroke	2 (3.1)	20 (6.7)	9 (3.0)	35 (8.1)
All-cause death	3 (4.7)	12 (4.0)	8 (2.7)	7 (1.6)
MI	0 (0)	0 (0)	1 (0.7)	2 (0.7)
MACE	1 (2.9)	18 (8.9)	7 (4.8)	26 (8.6)
**Group C2: simultaneous procedures**				
Post-OP Hospital stay in days (M; Q1–Q3)	8 (7-12)	12 (7-19)	11 (7-17)	10 (6-16)
Major stroke or death	4 (5.6)	4 (11)	11 (6.9)	11 (8.9)
Any stroke	2 (2.8)	3 (8.6)	7 (4.4)	4 (3.2)
All-cause death	4 (5.6)	3 (8.6)	8 (5.0)	8 (6.5)
MI	0 (0)	0 (0)	0 (0)	1 (1.3)
MACE	1 (2.2)	2 (8.0)	5 (5.9)	5 (6.6)
**Group C3: other indications**				
Post-OP Hospital stay in days (M; Q1–Q3)	5 (3-7)	5 (4-6)	5 (4-6)	5 (4-6)
Major stroke or death	9 (5.6)	6 (7.6)	15 (5.7)	20 (5.3)
Any stroke	6 (3.8)	8 (10)	12 (4.5)	18 (4.8)
All-cause death	7 (4.4)	2 (2.5)	5 (1.9)	7 (1.9)
MI	2 (2.0)	0 (0)	1 (0.7)	0 (0)
MACE	5 (4.9)	7 (15)	7 (5.0)	11 (4.8)

M = Median, Q1/3 = first/third quartile, MI and MACE: from 2013–2016. MI = myocardial infarction, MACE = major adverse cardiovascular event. Group A, asymptomatic cases; group B, symptomatic cases electively treated for amaurosis fugax, transitory ischemic attack, stroke or other symptoms; group C1, patients suffering crescendo-TIA or stroke-in-evolution; group C2, CEA or CAS in the context of simultaneous procedures (e.g., simultaneously performed coronary artery bypass operation, peripheral arterial reconstruction). Group C3, CEA or CAS for other indications (e. g. carotid aneurysm, symptomatic coiling, redo carotid procedures, tandem stenosis).

Supplemental Table S2: Secondary outcomes in patients treated with carotid artery stenting (CAS)

	Certified vascular centre			
	Yes		No	
Certified stroke-unit	Yes	No	Yes	No
**Group A: asymptomatic**				
Post-OP Hospital stay in days (M; Q1–Q3)	2 (2-3)	2 (2-3)	2 (1-4)	2 (2-3)
Major stroke or death	1 (0.3)	1 (0.5)	21 (2.4)	14 (1.4)
Any stroke	3 (0.8)	1 (0.5)	31 (3.5)	20 (1.9)
All-cause death	0 (0.0)	0 (0.0)	5 (0.6)	5 (0.5)
MI	0 (0.0)	0 (0.0)	0 (0.0)	0 (0.0)
MACE	0 (0.0)	1 (0.9)	17 (3.7)	8 (1.2)
**Group B: symptomatic, elective**				
Post-OP Hospital stay in days (M; Q1–Q3)	3 (2-6)	3 (2-6)	3 (2-6)	3 (2-5)
Major stroke or death	5 (2.8)	3 (3.5)	21 (2.3)	11 (2.9)
Any stroke	5 (2.8)	3 (3.5)	26 (2.9)	14 (3.7)
All-cause death	2 (1.1)	1 (1.2)	8 (0.9)	4 (1.1)
MI	0 (0.0)	0 (0.0)	1 (0.2)	0 (0.0)
MACE	3 (3.1)	1 (2.2)	19 (4.1)	5 (2.3)
**Group C1: symptomatic, emergency**				
Post-OP Hospital stay in days (M; Q1–Q3)	9 (5-13)	8 (5-10)	8 (5-13)	4 (3-10)
Major stroke or death	7 (9.9)	7 (14)	37 (10)	6 (9.2)
Any stroke	7 (9.9)	4 (8.2)	13 (3.6)	4 (6.2)
All-cause death	3 (4.2)	6 (12)	30 (8.2)	2 (3.1)
MI	1 (1.9)	0 (0.0)	0 (0.0)	0 (0.0)
MACE	7 (13)	2 (6.7)	3 (1.7)	3 (8.6)
**Group C2: simultaneous procedures**				
Post-OP Hospital stay in days (M; Q1–Q3)	10 (7-15)	8 (4-13)	7 (3-12)	5 (2-12)
Major stroke or death	4 (6.0)	6 (19)	24 (11)	5 (6.8)
Any stroke	2 (3.0)	5 (16)	14 (6.3)	4 (5.5)
All-cause death	3 (4.5)	5 (16)	21 (9.4)	4 (5.5)
MI	0 (0.0)	0 (0.0)	0 (0.0)	0 (0.0)
MACE	2 (5.6)	1 (11)	5 (5.9)	2 (5.6)
**Group C3: other indications**				
Post-OP Hospital stay in days (M; Q1–Q3)	5 (2-10)	11 (7-13)	5 (2-11)	3 (2-8)
Major stroke or death	5 (12)	2 (6.5)	17 (8.6)	2 (4.4)
Any stroke	5 (12)	2 (6.5)	8 (4.1)	3 (6.7)
All-cause death	5 (12)	0 (0)	13 (6.6)	1 (2.2)
MI	0 (0.0)	0 (0.0)	0 (0.0)	1 (4.2)
MACE	2 (8.0)	1 (4.2)	5 (4.6)	2 (8.3)

M = Median, Q1 = first quartile, Q3 = third quartile, MI and MACE: from 2013-2016. MI = myocardial infarction, MACE = major adverse cardiovascular event. Group A, asymptomatic cases; group B, symptomatic cases electively treated for amaurosis fugax, transitory ischemic attack, stroke or other symptoms; group C1, patients suffering crescendo-TIA or stroke-in-evolution; group C2, CEA or CAS in the context of simultaneous procedures (e.g., simultaneously performed coronary artery bypass operation, peripheral arterial reconstruction). Group C3, CEA or CAS for other indications (e. g. carotid aneurysm, symptomatic coiling, redo carotid procedures, tandem stenosis).

Supplemental Table S3: Regional characteristics of treating hospitals.

	**Certified vascular centre**			
	Yes		No	
**Certified stroke-unit**	Yes	No	Yes	No
**Socio-economic Indices (M, Q1/3)**				
UX	0.79 (0.44-1.27)	1.13 (0.64-1.81)	0.48 (-0.26-1.15)	0.32 (-0.23-1.10)
SGX	-0.68 (-1.08- -0.35)	-0.48 (-0.92 – -0.01)	-0.70 (-1.08 – -0.05)	-1.09 (-1.45- -0.55)
GISD	0.35 (0.14-0.40)	0.30 (0.17-0.38)	0.36 (0.26-0.41)	0.36 (0.27-0.42)
**Outpatient requirement planning (headcount)**				
Vascular surgeons	3 (3-14)	3 (3-8)	3 (0-3)	3 (3-3)
Cardiologists / Angiologists	8 (3-106)	10 (4-30)	6 (3-9)	3 (3-11)
Neurologists	12 (6-73)	11 (3-27)	3 (3-7)	3 (3-12)
Neurologists / Psychiatrists	3 (3-17)	3 (3-12)	3 (3-3)	3 (3-3)
Family doctors	113 (45-1116)	112 (54-380)	81 (49-105)	88 (68-167)
**Reachability (by car, minutes)**				
Intermediate level settlement (Mittelzentrum)	0 (0-0)	0 (0-0)	0 (0-8)	6 (0-9)
Main towns (Oberzentrum)	0 (0-0)	0 (0-0)	0 (0-17)	14 (0-20)
**In-patient and out-patient healthcare structures***				
Density of family doctors	74 (72-82)	70 (69-72)	70 (65-74)	69 (63-73)
Density of hospital beds	1416 (799-1877)	1063 (729-1794)	873 (672-1501)	584 (353-867)
Density of hospitals	3.5 (3.3-5.8)	3.4 (2.6-5.5)	3.4 (2.4-4.8)	3.2 (2.2-3.9)
Density of hospital beds (vascular surgery)	7.4 (0-44.4)	13.2 (0-34.5)	6.9 (0-42.9)	0 (0-7.9)
Density of hospital beds (neurology)	90.2 (38.6-105.5)	37.0 (19.2-71.8)	48.5 (32.2-106.8)	18.9 (0-39.1)
Density of certified stroke units	1.4 (0.5-1.6)	0.2 (0-1.3)	0.9 (0.7-1.6)	0 (0-0.3)
Density of certified vascular centres	1.3 (0.5-1.4)	1.3 (0.3-1.4)	0 (0-0)	0 (0-0)

M = Median, Q1 first quartile, Q3 = third quartile, UX = Urbanitätsindex, SGX = Sozioökonomischer Gesundheitsindex, GISD = standardised German Index of Socioeconomic Deprivation. * = per 100 000 inhabitants

Supplemental Figure S1: Patient flowchart

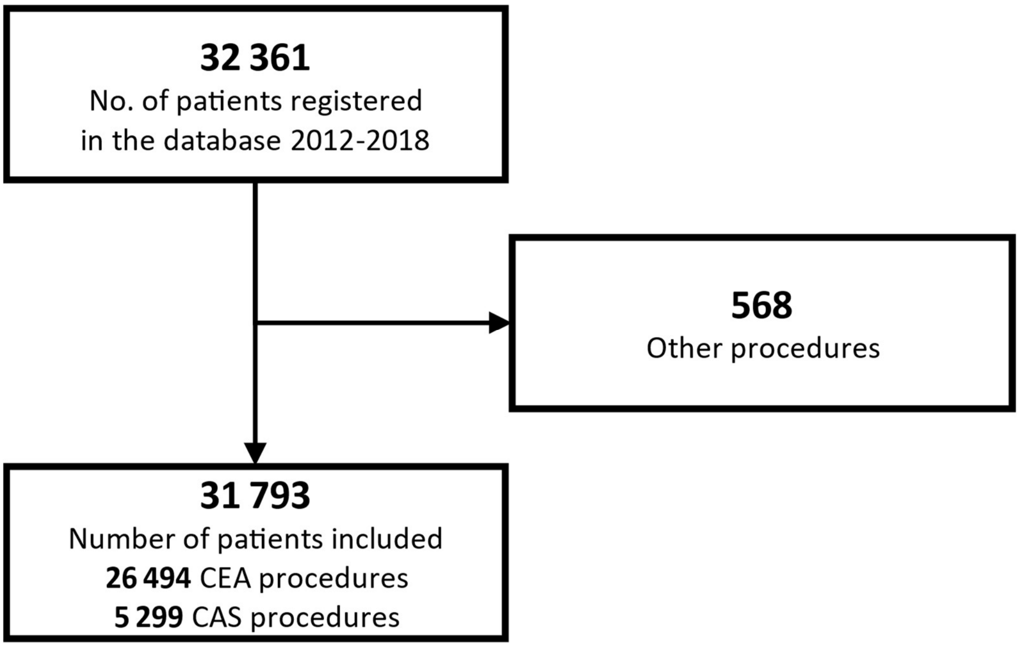

Please see methods section for detailed definition of inclusion and exclusion criteria.
